# Independently outgrowing neurons with a geometric synapse formation model develop realistic network connectivity patterns with small-world properties

**DOI:** 10.1186/1471-2202-12-S1-P356

**Published:** 2011-07-18

**Authors:** Andrew Carnell, Sander de Ridder, Jaap van Pelt, Arjen van Ooyen

**Affiliations:** 1 Integrative Neurophysiology, Center for Neurogenomics and Cognitive Research (CNCR), VU University Amsterdam, De Boelelaan 1085, 1081 HV Amsterdam, The Netherlands

## 

In neural networks, connectivity plays an important role in cognition, while the geometry of neuronal arborisation is an important determinant of connectivity. However, precisely how synaptic connectivity depends on the detailed morphology of neurites remains poorly understood. The aim of this paper is to study the connectivity patterns of neural networks comprising neurons with realistic morphologies. These networks are created with our modelling tool NetMorph [1], a simulation framework that simulates neuronal morphogenesis from the perspective of the individual growth cone in a stochastic phenomenological manner. NetMorph has been shown to create realistic neuronal morphologies, [1].

The growth of networks of layer 2/3 neurons was simulated using NetMorph. A realistic minimum neuron separation of 20 micron was used. The method used for synapse formation combines the proximity of axon and dendrite line-pieces [2] with a `crossing criterion', [3].

A suite of various connectivity measures was used to analyse emergent network connectivity including analysis of synaptic distributions, small-world properties and of specific network *shape parameters*. Small-world topologies have been observed in numerous brain regions and allow for segregated as well as integrated processing. They are associated with both high local and high global efficiency and have low wiring cost and this makes them attractive models for the brain. It is shown that small-world connectivity is observed in all analysed NetMorph generated networks. The work in this paper demonstrates that shape parameters, such as Euclidean distances from synaptic locations to postsynaptic somata, produce distributions that are highly similar to those distributions produced by experimentally observed networks. Our preliminary findings indicate that realistic neuronal morphologies, simple geometry-based synapse formation rules and independently developing neurons are capable of producing networks with realistic synaptic distributions, connectivity patterns and small-world properties.
